# Causal Impact of Hemoglobin Levels on Global Quality of Life in Patients with Cancer

**DOI:** 10.3390/jcm15041579

**Published:** 2026-02-17

**Authors:** Mustafa Serkan Alemdar, Hakan Sat Bozcuk

**Affiliations:** 1 Department of Medical Oncology, Istinye University, 34396 Istanbul, Türkiye; 2 Department of Medical Oncology, Medical Park Hospital, 07160 Antalya, Türkiye; 3 Private Oncology Practice, 07160 Antalya, Türkiye; hbozcuk@gmail.com

**Keywords:** causal inference, quality of life, cancer, machine learning, random forest classifier, anemia

## Abstract

**Background:** Current cancer treatment strategies target the preservation and enhancement of patient quality of life. Therefore, we aimed to assess the causal impact of hemoglobin (Hb) levels on global quality of life in cancer patients. **Methods:** We conducted a retrospective analysis using data collected from new cancer patients. The dataset included responses to the European Organization for Research and Treatment of Cancer Quality of Life Questionnaire, demographic, and disease-related variables. We applied the Linear Non-Gaussian Acyclic Model (LiNGAM) algorithm to identify potential causal relationships among those, and their impact on global quality of life. Furthermore, to evaluate the relative importance of Hb levels on global quality of life, we utilized a Random Forest Classifier (RFC). **Results:** The Random Forest Classifier (RFC) emerged as the most accurate model for classifying global quality of life scores (QL2) in our analysis. RFC analysis showed that Hb level ranked as the 10th most important feature among the 23 predictors in all patients for the global quality of life. However, the LiNGAM algorithm identified the Hb value as the most significant causal factor on global quality of life (total causal effect = 3.5) for anemic patients. Moreover, the Hb value was also a significant causal factor in patients in the “other cancers” and “stage 4” subcategories, with total causal effect figures of 2.9 and 2.5, respectively. **Conclusions:** This study suggests that Hb levels may exert a beneficial causal effect on global quality of life in cancer patients with anemia and among those with stage 4 disease and cancers other than breast, lung, or colorectal cancer.

## 1. Introduction

Anemia is a common comorbidity in cancer patients, often resulting in secondary complications. According to the European Cancer Anemia Survey (ECAS) report, anemia is defined as a hemoglobin (Hb) level below 12 g/dL. Using this cutoff, the survey report found that the prevalence of anemia among cancer patients was 39.3% [1]. Similarly, a study examining the prevalence of anemia among hospitalized cancer patients during two distinct time periods found rates ranging from 16% to 26% [2].

Anemia can significantly impair daily functioning and overall well-being in cancer patients. Several studies have examined the relationship between anemia and its effects on quality of life, cognitive function, and depression in cancer patients [3,4,5,6]. For example, Chung et al. found a correlation between low Hb levels and depression in patients with late-stage cancer [3]. Another study examined the incidence of postoperative cognitive dysfunction in 170 cancer patients who had undergone surgery. The findings revealed that anemia is an independent risk factor for postoperative cognitive dysfunction observed three months post-surgery [4]. Additionally, a study utilizing the Functional Assessment of Chronic Illness Therapy-Fatigue (FACIT-F) questionnaire to measure quality of life found a direct correlation between Hb levels and quality of life scores. Specifically, an increase of 1 g/dL in Hb levels was found to lead to a notable improvement in quality-of-life scores [6].

In recent years, artificial intelligence (AI) has become more prominent in medical data analysis. It offers new ways to evaluate data without bias, identify the effects of complex and often unpredictable variables, and generate decision trees based on the analysis. AI has also been extensively used to study quality of life in medical oncology research [7]. For example, a machine learning model was used to evaluate the decrease in quality of life experienced by 286 thyroid cancer patients after thyroidectomy. This model identified specific features that influenced quality of life outcomes [7]. In another study, Pinto et al. applied a random forest model to assess the quality of life in melanoma patients and concluded that random forest regressions provide valuable support for clinical and rehabilitation strategies [8].

Artificial intelligence modeling was employed to evaluate quality of life across various cancer groups. However, in conjunction with this approach, we specifically aimed to test the causal impact of Hb values, in general, or in subgroups of cancer patients, on quality of life. Thus, we hypothesized that Hb level has a causal, positive effect on global quality of life (QL2) in patients with cancer, and that this effect would be most pronounced in patients with anemia and in clinically relevant subgroups, such as advanced-stage disease. We further hypothesized that while Hb may show only modest predictive importance in the overall cohort when considered alongside multiple clinical and quality-of-life variables, causal modeling would identify Hb as an important driver of QL2 in selected subpopulations. To test these hypotheses, we combined machine-learning with causal inference in the full cohort and predefined subgroups.

## 2. Material and Methods

### 2.1. Selection of Cases and Data Collection Process

This study is an extension of the initial audit phase, during which the Turkish version of the EORTC QLQ-C30 version 3 questionnaire was provided to all cancer patients visiting a private hospital’s cancer center and under the care of the same attending physician at their first appointment. Data were collected from all consecutive new patients who consented to complete the questionnaire between January 2018 and July 2021. Additionally, primary demographic and disease-related information was gathered. Quality-of-life dimension scores, including global quality of life scores (QL2), were calculated according to the outlined guidelines (https://www.eortc.org/app/uploads/sites/2/2018/02/SCmanual.pdf, accessed on 3 December 2024). The Medical Park Hospital Ethics Committee in Antalya approved this study on 17 January 2024 under reference number 2024/1.

In the second phase of the study, we obtained Hb values for each patient at the time they completed the questionnaire by reviewing their medical records. The dataset was expanded with additional cases while excluding cases with missing values for any of the 23 predictor variables, including Hb levels. The final database only included cases with complete data to optimize the reliability and robustness of the analysis. The Medical Park Hospital Ethics Committee in Antalya approved this phase of the study on 18 January 2024 under reference number 2024/2.

For this study, we collected data including Hb values, EORTC quality of life data, and demographic variables such as age, gender, type and stage of cancer, active treatment status, time since diagnosis, Eastern Cooperative Oncology Group (ECOG) performance status, and presence or absence of anemia. Although the ECAS guidelines define anemia as an Hb value below 12 g/dL, we wanted to account for gender-specific Hb values. For this study, anemia was defined as an Hb value below 13 g/dL in men and below 12.0 g/dL in women, according to the World Health Organization (WHO) recommendations [1,9]. All code for data analysis in this project was written in the Python 3 programming language, and the Google Colab service was chosen as the coding environment [10].

### 2.2. Assessment of Feature Importance

We dichotomized QL2 around the median value into good (global quality of life score ≥59) or poor (global quality of life score <59) quality of life categories. We then used a list of machine learning classifier algorithms from the PyCaret library to find the most accurate algorithm for our task, which was the classification of QL2 [11]. Random Forest Classifier (RFC) was the top performer and was used for this analysis. Other algorithms considered included Extra Trees Classifier, Ridge Classifier, Linear Discriminant Analysis, Naive Bayes, Logistic Regression, Light Gradient Boosting Machine, Extreme Gradient Boosting, Ada Boost Classifier, Gradient Boosting Classifier, SVM—Linear Kernel, K Neighbors Classifier, Decision Tree Classifier, Quadratic Discriminant Analysis, and Dummy Classifier. Feature importance was assessed taking into account the Variable Importance Scores using the RFC classifier. The RFC classifier was obtained through the open source PyCaret Python library, available at https://github.com/pycaret/pycaret accessed on 3 December 2024.

**Figure 1:**
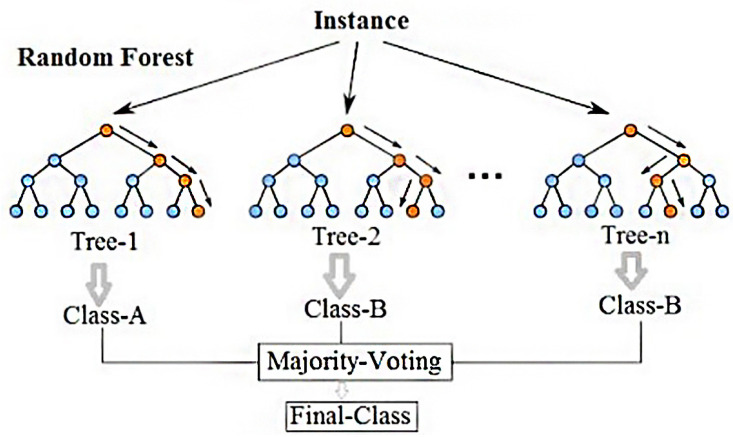
Random Forest Classifier.

An RFC is an ensemble machine learning method employed for classification tasks [12]. It operates by constructing a multitude of decision trees, each trained on a random subset of features and data points. Predictions are made by aggregating the votes of each individual tree, resulting in a more robust and accurate classification compared to a single decision tree. This approach also reduces the risk of overfitting the model to the training data. RFCs are known for their versatility in handling various data types and their ability to handle missing values effectively. See Figure 1 for a representation of the RFC algorithm.

### 2.3. Assessment of Causality

To evaluate the plausibility of LiNGAM’s non-Gaussianity assumption, we assessed the distribution of continuous variables using the Shapiro–Wilk test and computed skewness and kurtosis values. Normality testing was not applied to binary/ordinal variables.

To assess causal relationships, we utilized the LiNGAM Python library to implement a fully automated machine learning approach based on the Linear Non-Gaussian Acyclic Model (LiNGAM) [13]. This allowed us to visualize the LiNGAM adjacency matrix and calculate the total causal effects of various factors. In particular, we concentrated on the causal effect of Hb level on the global quality of life score (QL2; interval scale) in the overall population or in subgroups of interest with different types of cancer, advanced versus earlier stages, who receives versus does not receive active treatment, and who does or does not have anemia. Thus, the total causal effect of Hb on QL2 was calculated separately in subgroups of patients with breast cancer, lung or colorectal cancers, other cancers, stage 4 disease, stages 1–3 disease, active therapy, no active therapy, anemia, and no anemia. To align with the requirements of each analytic framework we utilized in this study, QL2 was dichotomized for machine-learning classification as stated above, facilitating an evaluation of predictive performance and feature-importance ranking across models. Conversely, QL2 was modeled as a continuous variable in the LiNGAM causal analysis to quantify causal effects and derive the causal importance of the predictors.

**Figure 2:**
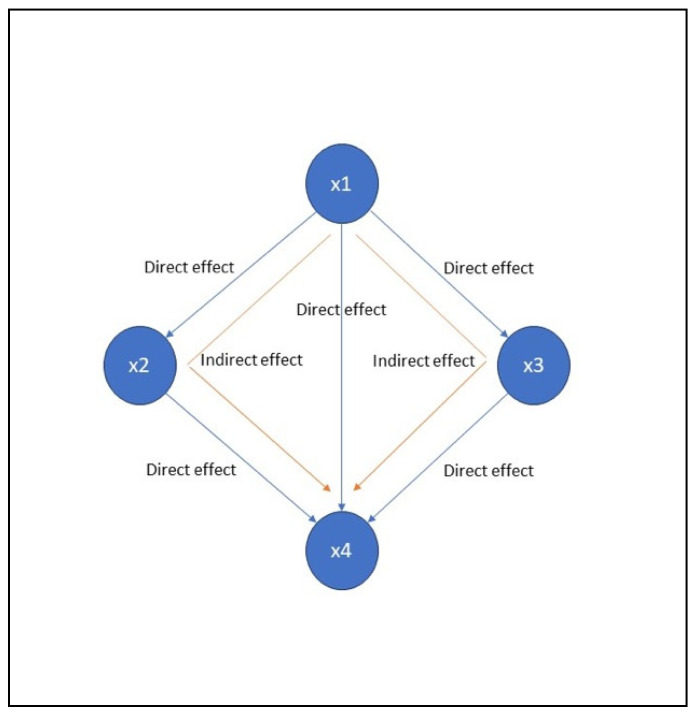
Causal effects in LINGAM. x1, x2, x3, and x4 are the variables in a causal network diagram.

The LiNGAM machine learning method is adept at uncovering causal relationships within observational data, particularly when the data exhibit non-Gaussian distributions [13]. Unlike traditional techniques that rely solely on covariance structures, LiNGAM leverages the non-Gaussianity to differentiate causal effects from mere correlations. Additionally, it operates under the assumption of acyclicity, meaning that the causal relationships do not form any closed loops. This allows LiNGAM to identify the direction of causality between variables, providing valuable insights into the underlying mechanisms at play. In our study, we assessed the directionality and the magnitude of the causal relationship of Hb level on QL2 by means of the total causal effect, which takes into account both the direct and indirect causal effects. These effects, as they are assessed in LiNGAM, are plotted in Figure 2.

In this manuscript, the authors used Chat GPT 4.0 and Gemini to improve readability. After using these tools, the authors reviewed and edited the content.

## 3. Results

### 3.1. General Features

A total of 382 cases were included in this study. The mean (SD) age was 54.80 (13.61) years, and the majority were female (61.3%), breast cancer diagnosis (38.5%), and a mean (SD) stage of 2.71 (1.15). The mean (SD) Hb value was 12.79 (1.71) g/dL, and 41% of our subjects had anemia. The mean (SD) QL2 figure was 56.55 (25.38) out of a scale of 100. These general characteristics can be viewed in Table 1.

Normality was rejected for 16 out of 17 continuous variables in our dataset (all with Shapiro *p* values < 0.001), indicating substantial departures from Gaussianity. One of the 17 continuous variables (Hb) was marginally normally distributed; “not strongly non-normal” with Shapiro W = 0.993, Shapiro *p* = 0.083, demonstrating a left skewness of −0.201 and slightly heavier tails with an excess kurtosis of 0.322.

### 3.2. Feature Importance for Hb Levels on Global Quality of Life

In order to differentiate between good (global quality of life score ≥59) and poor (global quality of life score <59) quality of life categories, a total of 15 machine learning classification models were coded and compared in terms of accuracy. RFC was selected to be the most accurate model with an initial accuracy figure of 0.75, among others. See Table 2 for a comparison of model accuracies. In this table, 15 different machine learning models were compared in terms of their performance parameters.

With hyperparameter optimization, the accuracy figure of the RFC model improved to 0.88, and the area under curve (AUC) value was 0.96, consistent with an accurate classifier performance. The automatically tuned and optimized RFC model had a maximum depth of 6, “min_impurity_decrease” of 0.001, “min_samples_leaf” of 6, “min_samples_split” of 9, and “*n*_estimators” of 190. This RFC technique was employed and selected 19 out of the 23 predictors available to achieve a Recursive Feature Elimination with Cross-Validation (RFECV) score of 0.76. This step yielded a more efficient and interpretable model with a smaller number of predictors [14].

**Figure 3:**
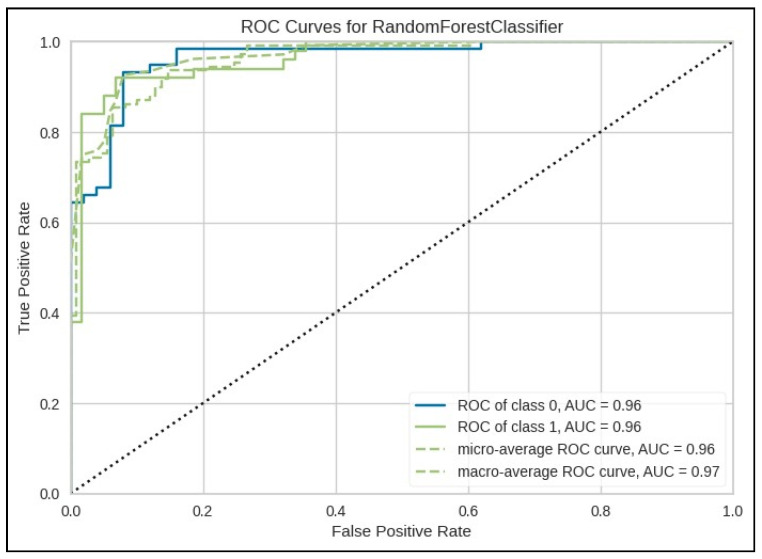
AUC plot for the Random Forest Classifier to predict global quality of life.

Figure 3 presents the AUC plot for the RFC, demonstrating the model’s strong classification performance. The ROC value for both groups was 0.96 each.

**Figure 4:**
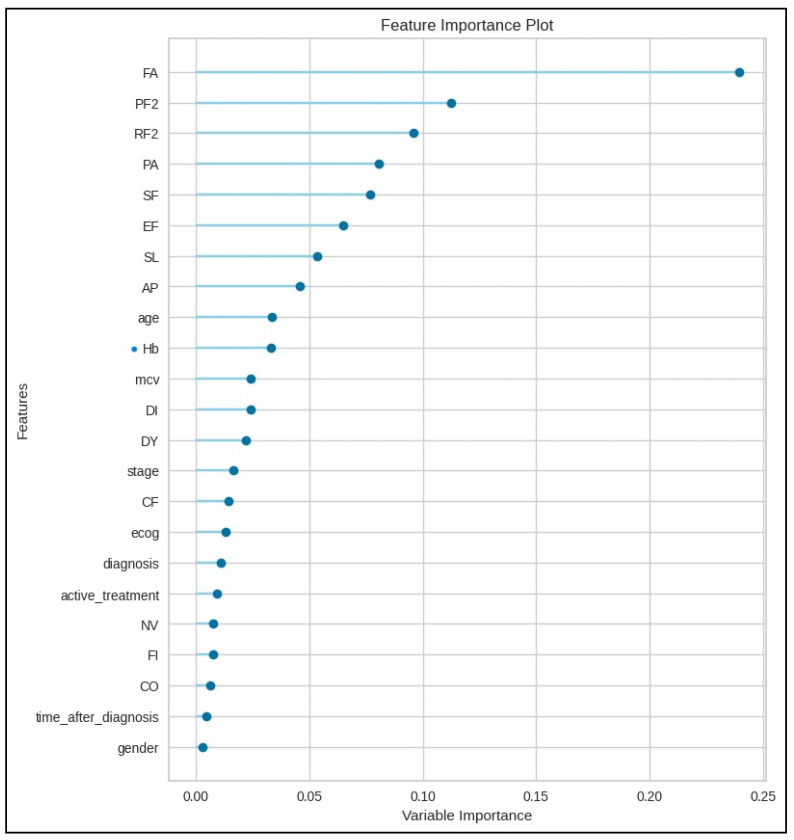
The feature importance of Hb level on the global quality of life in all patients. Abbreviations and explanations: feature importance on global QoL; QL2, PF2; physical functioning, RF2; role functioning, EF; emotional functioning, SF; social functioning, CF; cognitive functioning, FA; fatigue, NV; nausea and vomiting, PA; pain, DY; dyspnea, SL; insomnia, AP; appetite loss, CO; constipation, DI; diarrhea, FI; financial difficulties, Hb; hemoglobin, mcv; mean corpuscular volume, stage; TNM stage of cancer, ecog; ECOG performance status score, diagnosis; type of cancer, time_after_diagnosis; number of months that elapsed after the diagnosis until completion of the EORTC QLQ-C30 questionnaire.

The resultant RFC model was then utilized to rank the feature importance of the predictors evaluated in this study, particularly to assess the relative importance of Hb levels on global quality of life scores. As seen in Figure 4 below, taking into consideration the associated feature importance values, Hb levels ranked 10th among the 23 predictors evaluated by the RFC model when all cases in the study were considered.

### 3.3. Causal Impact of Hb Levels on Global Quality of Life

When the LiNGAM method was run for all cases, the Hb level was not shown to exert any causal effect on global quality of life, with a total causal effect figure of 0. However, for the anemic cases, Hb level was the most significant causal factor on global quality of life, with a total causal effect of 3.5, demonstrating a high beneficial causal impact. For the anemic cases, again, there were 9 fewer causally influential factors on global quality of life with total causal effect figures ranging from −0.6 to 0.4, and these factors were the scores of various symptom and functional subscales of EORTC QLQ-C30. Likewise, in different patient subpopulations (other cancers, active treatment, no active treatment, stage 4) from our cohort, Hb level was found to possess causal influence on global quality of life, with total causal effect figures ranging from 2.5 to 2.9. Refer to Table 3 for the total beneficial causal effect of Hb levels on global quality of life in all cancer patients and in various prespecified subpopulations. These subpopulations included patients with breast cancer, lung or colorectal cancers, other cancers, stage 4 disease, stages 1–3 disease, active therapy, no active therapy, anemia, and no anemia.

## 4. Discussion

In our study, Hb level was identified as the most causally impactful feature influencing quality of life in the anemic subgroup, with a total causal effect of 3.5. However, when analyzing all cancer patients from a correlative perspective, Hb level ranked as the 10th most influential feature associated with quality of life. Notably, the nine features that ranked higher in the feature importance analysis consisted of various quality of life subdomain scores and patient age. A meta-analysis of five randomized studies supports the importance of Hb levels. These studies reported significant improvements in FACIT-F scores among anemic cancer patients following an increase in Hb levels [15]. The meta-analysis also showed that increasing Hb levels by more than 2 g/dL was linked to a significant improvement in FACIT-F scores [15]. Moreover, a study evaluating the impact of anemia on quality-of-life scores in 133 chemotherapy patients emphasized the critical role of Hb levels in alleviating functional and physical symptoms commonly associated with cancer-related fatigue [16]. In another study involving 449 cancer patients, Animaw et al. identified anemia as a significant risk factor for cancer-related fatigue. They reported that anemic patients were 2.01 times more likely to experience fatigue than their non-anemic counterparts [17]. These findings collectively underscore the pivotal role of Hb levels in improving quality of life and alleviating cancer-related symptoms, especially in patients with anemia. Our results align with existing literature underscoring the strong relationship between Hb levels and quality of life. However, our study goes further than previous research by providing evidence that supports a causal relationship between Hb levels and global quality of life. Using the LiNGAM causal modeling methodology, we provide insights into potential causal pathways linking Hb levels to quality of life. Our findings complement and extend the current body of evidence.

Additionally, we demonstrate that Hb level is a significant factor influencing the quality of life of patients with cancers other than breast, lung, or colorectal cancers. The total causal effect was 2.9. Numerous studies have reported an association between Hb levels and quality of life in patients with breast and colorectal cancers. However, other studies, including the present one, have not been able to confirm this relationship [15,16,17,18,19]. For instance, a study of 130 breast cancer patients identified anemia as one of the most commonly reported side effects of chemotherapy. However, the study did not find anemia to be a significant factor affecting the patients’ quality of life [18]. Similarly, a study investigating the impact of oral nutritional support on the quality of life of patients with colorectal cancer observed that improvements in Hb levels did not significantly affect quality-of-life outcomes [19]. To gain a comprehensive understanding of the relationship between Hb levels and quality of life (QoL) across different cancer types, a detailed analysis of factors specific to various cancers, such as symptom burden and fatigue levels, is crucial. These factors should be evaluated alongside commonly used treatment modalities and their associated side effects. Collectively, these factors may act as confounding variables when assessing the true impact of Hb levels on quality of life in different cancer types.

Finally, the results of our study indicate that Hb level is not a significant factor in determining quality of life in patients with stages 1–3 cancer. However, in patients with advanced-stage cancer, Hb level is a key factor in quality of life. We believe that this may be partially because advanced-stage cancer patients are more susceptible to anemia. The median FACIT-F score for 110 cancer patients receiving palliative care was 14. Among these patients, FACIT-F scores were significantly lower for those with Hb values below 10 g/dL [20]. In a study by Xi et al. that investigated the factors affecting performance status after chemotherapy using three different models, Hb level was found to be one of the six most effective factors [21].

What could be the role of anemia in shaping quality of life for patients with advanced cancer, and in cases with cancer diagnoses other than breast, lung and colorectal cancers? Anemia may significantly impact quality of life, particularly in patients with metastatic cancers, possibly where QoL is already compromised by multiple disease-related factors. In such patients, fatigue due to tumor burden and the use of cytotoxic chemotherapies can independently reduce QoL, and the presence of anemia may further exacerbate this decline. This effect could be more pronounced in metastatic cancers other than breast, lung, or colorectal cancer, where treatment options may involve agents with potentially higher toxicity profiles. In these settings, the cumulative impact of anemia alongside systemic symptoms and treatment-related adverse effects likely contributes more substantially to the deterioration of global quality of life.

This study focused on applying the LiNGAM algorithm to causal analysis. Using this methodology, we tested whether clinically intuitive or unexpected features were causally impactful. Unlike traditional machine learning models, LiNGAM is specifically designed to uncover causal relationships rather than mere associations. This provides deeper insights into the factors influencing the quality-of-life index. By identifying causal pathways, LiNGAM challenges previous assumptions and offers a more robust understanding of the underlying dynamics. This is particularly important in medical research, where establishing causality can inform targeted interventions and treatment strategies. In contrast, many studies in the literature have utilized machine learning models for association-based analyses. For example, Choe et al. employed three different machine learning models—random forest, support vector machine, and extreme gradient boosting—to evaluate factors affecting quality of life in 1005 cancer patients [22]. Similarly, another study used machine learning algorithms to analyze the impact of health-related quality of life scores on five-year survival in lung cancer patients, comparing the models’ performance through fivefold cross-validation [23]. While these studies provide valuable insights, they are limited to identifying predictive associations rather than causal relationships. To our knowledge, no prior studies have used causal modeling, such as LiNGAM, to explore the relationship between quality of life and anemia in cancer patients. For this reason, the use of LiNGAM is a significant strength of our study as it allows us to explore potential causal relationships beyond correlation. By employing this novel causal methodology, our research provides more actionable insights that could guide clinical decision-making and enhance patient outcomes, particularly in understanding how anemia directly impacts the quality of life in cancer patients.

This study has certain limitations. Firstly, the retrospective nature of the study and the relatively small sample size may affect the generalizability of the findings. Secondly, variables related to the underlying causes of anemia and its treatment were not included within the scope of this study, which could have provided additional insights into the broader context of anemia in cancer patients. However, some major confounders of the Hb–quality of life association may not have been fully accounted for in our analyses. Thirdly, also in relation to the predictor and confounder features we evaluated in this study, and given the intentional conceptual proximity between QL2 and other EORTC subscales, models including these subscales should be interpreted as within-instrument relationships, and both predictive importance and causal pathways may be influenced by construct overlap. Fourthly, subgroup LiNGAM analyses may be vulnerable to unstable estimates and chance findings due to smaller sample sizes and repeated stratification. Accordingly, subgroup-specific causal effects should be interpreted as hypothesis-generating and warrant confirmation in independent cohorts. Fifthly, because all participants were managed by a single physician at a single center, external validity may be limited, as patient mix, supportive care practices, and treatment pathways can differ across institutions. Therefore, our findings should be confirmed in multi-center cohorts with broader case-mix and care settings. Finally, some cases with missing predictor variables—primarily due to the unavailability of timely Hb measurements or ECOG performance assessments—were excluded from the analyses. Nevertheless, since these cases were few in number, it is unlikely that their exclusion had a significant impact on the main results of our study. Additionally, although some cases with missing key predictor variables were excluded prior to analysis to ensure data consistency, particularly for variables like Hb levels and ECOG scores, we note that RFC is inherently robust to missing data. This robustness supports the reliability of the model’s performance, but the initial exclusions were made to maintain a cleaner dataset and ensure fairness across models during comparative analysis.

Also, the interpretation of effect size should be cautious. Prior work suggests that changes of approximately 5–10 points on the EORTC QLQ-C30 reflect a small change, 10–20 a moderate change, and >20 a large change [24]. Accordingly, under a linear approximation, a total causal effect of 3.5 corresponds to an estimated approximately +3.5 QL2 points per +1 g/dL increase in Hb; thus, a +3 g/dL increase in Hb would translate to an ≈+10.5-point difference in QL2, which is of moderate magnitude and meets or exceeds the commonly used ≥10-point benchmark for clinically meaningful change. Nevertheless, clinical meaningfulness remains context-dependent, and this observational, model-based estimate should not be interpreted as the guaranteed effect of a specific intervention in an individual patient.

Despite these limitations, we believe that the study adds value in several ways. First, to our knowledge, it is the first to apply a causal inference framework to examine the relationship between Hb levels (and anemia) and global quality of life in patients with cancer. Second, by using the LiNGAM algorithm, we move beyond correlation-based analyses and estimate causal pathways linking Hb to QL2, which may better inform clinically actionable hypotheses. Third, we complement the causal analysis with machine-learning modeling to rank the relative importance of Hb among a broader set of clinical and patient-reported variables. Together, this combined approach provides a more comprehensive view of the Hb–quality of life relationship and illustrates how integrating causal modeling with predictive methods can strengthen supportive care research in oncology.

In conclusion, in the modeling framework employed in this study, Hb level was identified as one of the most significant causal factors influencing global quality-of-life scores in cancer patients with anemia. Furthermore, Hb levels were found to have a measurable causal effect on quality of life in patients with various types of cancer, including advanced-stage disease. Consequently, these findings suggest that careful monitoring of Hb levels and the appropriate management of anemia may improve patient comfort and quality of life. However, prospective studies are needed to confirm this effect.

## Figures and Tables

**Table 1:** Demographic characteristics of the patients.

Feature	*n*	%	Min–Max	Mean	SD
*Clinical features*					
Total	382	100			
Hemoglobin level (mg/dL)			7.2–17.6	12.79	1.71
Anemia status *					
Anemia	158	41.36			
No anemia	224	58.64			
Age			18–83	54.80	13.61
Sex					
Male	148	38.74			
Female	234	61.26			
Ecog status			0–4	0.82	0.81
Diagnosis					
Breast cancer	147	38.48			
Lung or colorectal cancer	94	24.61			
Other cancers	141	36.91			
Stage			1–4	2.71	1.15
Active treatment					
Yes	243	63.61			
No	139	36.39			
Time since diagnosis					
Less than 6 months	310	81.15			
6 to 12 months	24	6.28			
More than 12 months	48	12.57			
*Quality of life dimensions*					
QL2			0–100	56.55	25.38
PF2			0–100	73.35	24.10
RF2			0–100	74.56	29.47
EF			0–100	74.28	23.86
CF			0–100	86.04	18.59
SF			0–100	77.44	25.66
FA			0–100	35.63	26.52
NV			0–100	7.90	17.19
PA			0–100	28.49	28.25
DY			0–100	15.88	26.31
SL			0–100	27.40	32.84
AP			0–100	23.56	30.60
CO			0–100	16.58	26.76
DI			0–100	10.30	21.16
FI			0–100	21.73	28.41

Quality of life dimensions: QL2; global quality of life, PF2; physical functioning, RF2; role functioning, EF; emotional functioning, CF; cognitive functioning, SF; social functioning, FA; fatigue, NV; nausea and vomiting, PA; pain, DY; dyspnea, SL; insomnia, AP; appetite loss, CO; constipation, DI; diarrhea, FI; financial difficulties. *; Anemia is defined as a hemoglobin value of less than 13.5 mg/dL in men, and less than 12.0 mg/dL in women. SD; standard deviation.

**Table 2:** Comparison of Machine Learning Models.

Model	Accuracy	AUC	Recall	Precision	F1	Kappa	MCC	TT (Sec)
Random Forest Classifier (rf)	0.7485	0.8298	0.753	0.7217	0.7315	0.4947	0.5018	0.212
Extra Trees Classifier (et)	0.7482	0.8131	0.7273	0.7329	0.7234	0.4922	0.4991	0.204
Ridge Classifier (ridge)	0.7445	0.8126	0.7894	0.7039	0.7408	0.4905	0.4987	0.037
Linear Discriminant Analysis (lda)	0.7445	0.8114	0.7894	0.7039	0.7408	0.4905	0.4987	0.062
Naive Bayes (nb)	0.7442	0.831	0.8371	0.6921	0.7529	0.4927	0.512	0.036
Logistic Regression (lr)	0.7403	0.8064	0.722	0.7329	0.7187	0.4783	0.4877	0.999
Light Gradient Boosting Machine (lightgbm)	0.7209	0.7838	0.7038	0.709	0.7013	0.4395	0.445	0.271
Extreme Gradient Boosting (xgboost)	0.7137	0.7974	0.7189	0.6975	0.6997	0.4266	0.4352	0.085
Ada Boost Classifier (ada)	0.7128	0.7609	0.7136	0.6982	0.6936	0.4245	0.4371	0.151
Gradient Boosting Classifier (gbc)	0.7095	0.7752	0.6939	0.6923	0.6852	0.4157	0.4226	0.309
SVM—Linear Kernel (svm)	0.7082	0.8234	0.5106	0.7413	0.578	0.4006	0.4373	0.037
K Neighbors Classifier (knn)	0.7049	0.7799	0.753	0.6631	0.6983	0.411	0.4235	0.055
Decision Tree Classifier (dt)	0.6902	0.6884	0.6614	0.6681	0.6568	0.376	0.3825	0.036
Quadratic Discriminant Analysis (qda)	0.6894	0.7711	0.6439	0.6792	0.6544	0.3731	0.3806	0.038
Dummy Classifier (dummy)	0.5354	0.5	0.0	0.0	0.0	0.0	0.0	0.035

MCC; Matthews Correlation Coefficient, TT; training time.

**Table 3:** Causal significance of hemoglobin levels on global quality of life.

Patient Subgroup	Total Causal Effect of Hb * on QL2 **	Effect Ranking ***	Number of Causally Significant Factors #
Overall	0	0	13
*Diagnosis*			
Breast cancer	0	0	1
Lung or colorectal cancer	0	0	0
Other cancer	2.9	2	4
*Stage*			
Stage 4	2.5	4	4
Stage 1 to 3	0	0	13
*Treatment status*			
Active therapy	2.8	3	3
No active therapy	2.9	2	2
*Anemia status*			
Anemia	3.5	1	10
No anemia	0	0	12

* Hemoglobin level, ** Global quality of life score, *** Ranking of the total causal effect of Hb on QL2, among all causally significant factors for the particular subgroup, # Number of causally significant factors for the particular subgroup.

## Data Availability

All data generated or analyzed during this study are included in this article. Further inquiries can be directed to the corresponding author (M.S.A.).
